# The molecular mechanisms driving physiological changes after long duration space flights revealed by quantitative analysis of human blood proteins

**DOI:** 10.1186/s12920-019-0490-y

**Published:** 2019-03-13

**Authors:** Daria N. Kashirina, Andrew J. Percy, Liudmila Kh. Pastushkova, Christoph H. Borchers, Kirill S. Kireev, Vladimir A. Ivanisenko, Alexey S. Kononikhin, Eugene N. Nikolaev, Irina M. Larina

**Affiliations:** 1 Institute for Biomedical Problems – Russian Federation State Scientific Research Center of RAS, Moscow, Russia; 2 Genome British Columbia Proteomics Centre, University of Victoria, Victoria, BC Canada; 3 Yu.A.Gagarin Research and Test Cosmonaut Training Center, Star City, Moscow Region, Russia; 4 Institute of Cytology and Genetics of SB RAS, Novosibirsk, Russia; 5 Novosibirsk State University, Novosibirsk, Russia; 6 V.L. Talrose Institute for Energy Problems of Chemical Physics, Russian Academy of Sciences, Moscow, Russia; 7 Moscow Institute of Physics and Technology, Dolgoprudny, Moscow region, Russia; 8 Skolkovo Institute of Science and Technology, Skoltech, Moscow region, Russia

**Keywords:** Cosmonauts, Mass spectrometry, Blood proteins

## Abstract

**Background:**

The conditions of space flight have a significant effect on the physiological processes in the human body, yet the molecular mechanisms driving physiological changes remain unknown.

**Methods:**

Blood samples of 18 Russian cosmonauts who had conducted long-duration missions to the International Space Station were collected 30 days before launch and on the first and seventh days after landing.

**Results:**

A panel of 125 proteins in the blood plasma was quantitated by a well-established and highly regarded targeted mass spectrometry approach. This method involves the monitoring of multiple reactions in conjunction with stable isotope-labeled standards at the University of Victoria - Genome BC Proteomics Centre.

**Conclusions:**

Reduction of circulating plasma volume during space flight and activation of fluid retention at the final stage of the flight affect the changes in plasma protein concentrations present in the first days after landing. Using an ANOVA approach, it was revealed that only 1 protein (S100A9) reliably responded to space flight conditions. This protein plays an important role in the functioning of the endothelium and can serve as a marker for activation of inflammatory reactions. Concentrations of the proteins of complement, coagulation cascades, and acute phase reactants increase in the blood of cosmonauts as measured the first day after landing. Most of these proteins’ concentrations continue to increase by the 7th day after space flight. Similar dynamics are observed for proteases and their inhibitors. Thus, there is a shift in proteolytic blood systems, which is necessary for the restoration of muscle tissue and maintenance of oncotic homeostasis.

**Electronic supplementary material:**

The online version of this article (10.1186/s12920-019-0490-y) contains supplementary material, which is available to authorized users.

## Background

The peculiar features of space radiation and microgravity during space flight generate risk for the cardiovascular system (CVS) thus increasing the risks to human space exploration. Space flight has significant influence on cardiovascular system function due to fluid redistribution and modification mechanisms involved in the regulation of blood pressure. Endothelial cells are active functioning elements of the CVS that participate in the factors affecting adaptation to space flight. It was shown that human endothelial cells from the umbilical vein are highly sensitive to microgravity [1]. The endothelium carries out barrier functions and is involved in the regulation of vascular tone, hemostasis, immune response, migration of blood cells to the vascular wall, and synthesis of inflammation factors and inhibitors. Thus, endothelial dysfunction can lead to the development of cardiovascular diseases. This dysfunction could also be the cause of the cardiovascular responses in cosmonauts known as cardiovascular disadaptation. However, the molecular mechanisms of physiological changes after space flight remain unknown. Recently, genomic and proteomic approaches have received significant attention. Proteomic methods with biological databases open new opportunities to reveal proteins participating in adaptive and pathological processes [2]. We have already analyzed the same set of cosmonauts’ blood proteins [3]. There were three distinct groups of proteins: 1) proteins with post-flight protein concentrations remaining stable; 2) proteins whose concentrations recovered slowly; and 3) proteins whose concentrations recovered rapidly to their pre-flight levels. Now we reanalyze this data set to reveal the reason for the decreased concentrations of these proteins with subsequent correlation with states of water and electrolytic exchange in cosmonauts in the final flight period. The previous analysis was aimed at identifying proteins involved in the adaptation process to the conditions of terrestrial gravity, and mainly, the dynamics of proteins’ level recovery were examined by looking at samples collected on the seventh day, while in the current study, the mechanism of changes in protein concentrations from samples taken on the first day after flight was examined.

It is well known, that plasma volume is reduced during spaceflight. Weightlessness exerts substantial effects on fluid redistribution in the human body. Plasma volume reduces by approximately 10–17% within the first day of spaceflight and this level is maintained until the end of mission [4, 5]. After returning to normal gravity cardiac stroke volume and arterial pressure falls below pre-flight upright levels [6]. It is necessary to restore initial plasma volume so fluid-retaining systems become activated. The volume of circulating blood increases due to modulation of hormones regulating water-salt metabolism, but the synthesis of blood proteins lags behind. Thus, plasma ‘dilution’ occurs where the blood contains less proteins compared with preflight data. In this manuscript, we analyzed results with a correction on plasma ‘dilution.’

## Methods

### Blood collection

Whole blood samples from eighteen Russian cosmonauts (all male, mean ± SD age: 44 ± 6 years old) were analyzed. All subjects provided written informed consent to participate in the experiment «Proteome» in advance of their long duration (169–199 days) missions on the ISS. The experiment «Proteome» was approved by Biomedicine Ethics Committee of the Russian Federation Scientific Research Center-Institute of Biomedical Problems, Russian Academy of Sciences/Physiology Section of the Russian Bioethics Committee Russian Federation National Commission for UNESCO, and the Human Research Multilateral Review Board, NASA, Houston, TX, USA. Blood samples were collected 30 days before launch (L-30), and on the first (R + 1) and seventh (R + 7) days after landing. Blood was taken by venipuncture from a vein in the cubital fossa. Collection was done in commercial SARSTEDT-Monovette® tubes containing EDTA as the anticoagulant. Blood samples were centrifuged at 2000 g for 15 min at + 4 °C to remove plasma and the supernatant was frozen at − 80 °C until usage.

### Target peptide panel

The target panel for liquid chromatography-mass spectrometric multiple reaction monitoring-based (LC/MRM-MS) analysis comprised proteins functioning in extracellular fluid. The target proteins are classified as high-to-moderate abundance, spanning an approximate concentration range from 33 mg/mL to 44 ng/mL [7]. In the MRM-with-SIS-peptide quantitative approach, proteotypic peptides (usually tryptic) serve as molecular representatives of the target proteins. To help with compensation for matrix-induced suppression or variability in LC-MS performance, C-terminal 13C/15 N-labeled peptide analogues were used as internal standards (SIS-peptides). These were synthesized and purified at the University of Victoria - Genome BC Proteomics Centre. The CZE-derived purity of the 142 SIS peptides was 94.2%, on average [8].

### LC/MRM-MS analysis

All steps of solution and sample preparation, LC/MRM-MS parameters, are described in the article [3]. LC/MRM-MS analysis was performed with a Zorbax Eclipse Plus RP-UHPLC column on a 1290 Infinity UPLC system (all from Agilent Technologies) that was interfaced to a triple quadrupole mass spectrometer (Agilent 6490) via Agilent’s Jet Stream™ source, operated in the positive-ion ESI mode. Plasma tryptic digests were analyzed only once. The MRM data was visualized and examined with MassHunter Quantitative Analysis software (version B.07.00; Agilent). Quantitation was done using linear regression analysis, as described previously [8].

### Bioinformational analysis

Statistical analysis was made with Statistics 7 software. Molecular function, biological processes, and paths were analyzed using DAVID database, PANTHER database, web application AmiGO, and search engine PubMed. The molecular masses and properties of proteins were annotated according to the information available from the UniProt KB database.

## Results and discussion

We obtained a list of all plasma proteins for each cosmonaut. In our data set, 125 plasma proteins were detected and quantitated. Using software Statistics 7 we performed analysis of variance (ANOVA). In the result we identified 19 proteins with statistically significant differences (*p*-value< 0,05) between samples collected at L-30, R + 1, and R + 7 (Table 1).

**Table 1:** Proteins with statistically significant differences (*p*-value< 0,05) between points of blood collection

Protein name	Means, fmol/μl			p-values			МW (kDa)
	L-30	R + 1	R + 7	L-30 vs R + 1	R + 1 vs R + 7	L-30 vs R + 7	
78 kDa glucose-regulated protein	6.3	4.6	5.3	**0.031**	0.216	0.200	72.3
Alpha-2-HS-glycoprotein	134.2	80.3	86.3	**0.037**	0.794	0.215	39.3
Apolipoprotein A-II	5168.8	4188.9	4410.8	**0.015**	0.423	**0.038**	11.2
Apolipoprotein A-IV	1277.7	1002.1	1425.1	0.067	**0.003**	0.275	45.4
Apolipoprotein C-III	8.1	6.7	8.3	0.121	**0.031**	0.850	10.9
Beta-2-microglobulin	110.6	96.6	116.8	**0.024**	**0.004**	0.322	13.7
Cadherin-5	16.2	14.2	15.7	**0.044**	0.193	0.635	87.5
cDNA FLJ53327	1007.1	802.2	959.2	**0.012**	**0.048**	0.535	77.8
Cystatin-C	71.9	41.9	49	**0.014**	0.630	0.218	15.8
Fibronectin	121.8	100.9	110.9	**0.044**	0.145	0.267	262.6
Fibulin-1	139.9	129.2	163.8	0.213	**0.000**	**0.021**	77.2
Gelsolin	811.6	611.1	737.2	**0.001**	**0.005**	0.182	85.7
Insulin-like growth factor binding protein, acid labile subunit	155.6	153.5	142.5	0.703	**0.047**	**0.014**	66.1
Lumican	510.3	446.9	528.2	**0.028**	**0.001**	0.503	38.4
Mannan-binding lectin serine protease 1	67.4	59.5	56.3	0.063	0.459	**0.010**	79.2
Neuropilin-2	42.7	41.1	46.8	0.432	**0.016**	0.106	104.9
Plasma serine protease inhibitor (PAI-3)	89.9	91.8	104.7	0.783	**0.045**	0.079	45.7
Protein S100-A9	2.7	6.2	3.8	**0.039**	0.070	0.152	13.2
Serotransferrin	9743.3	8235.4	8343.3	**0.028**	0.843	**0.030**	77.1

Note: *p*-values< 0,05 are bold

**Fig. 1:**
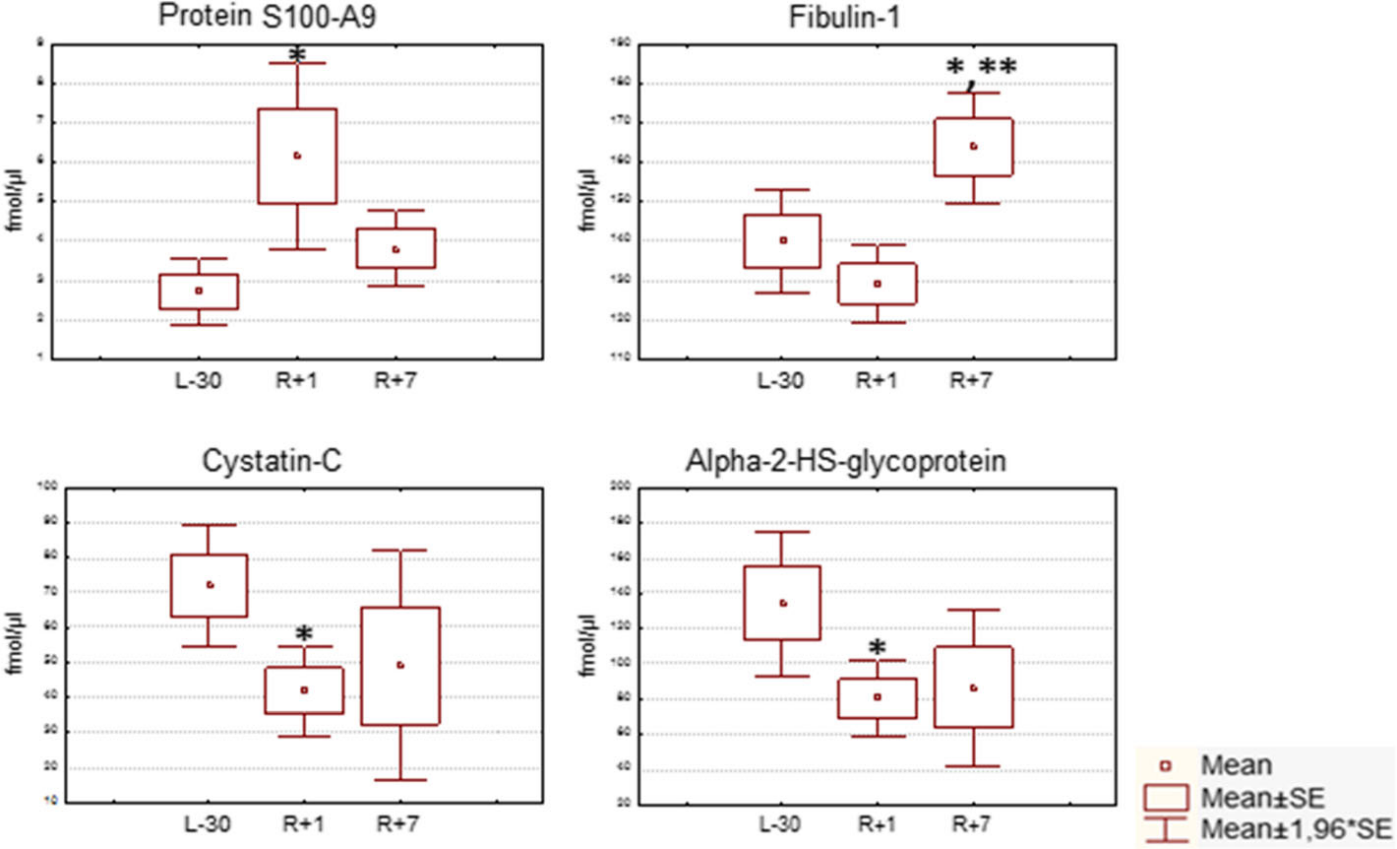
The dynamics of changes in concentrations of protein S100-A9, fibulin-1, cystatin-C, and alpha-2-HS-glycoprotein at the points of blood collection: 30 days before launch (L-30), and on the first (R + 1) and seventh (R + 7) days after landing. Notes to figure: *-statistically significant difference (*p*-value< 0,05) compared with L-30; ** - statistically significant difference (p-value< 0,01) compared with R + 1

Most of these proteins are decreased at R + 1 except PAI-3 (slight increase) and protein S100-A9 (significant increase) (Fig. 1 and Additional file 1: Figure S1). Myeloid-related protein S100A9 is a calcium- and zinc- binding protein, which plays a prominent role in the regulation of inflammatory processes and immune response. It is predominantly found as calprotectin (S100A8/A9), which has a wide array of intra- and extracellular functions. Calprotectin is predominantly expressed in myeloid cells. Its extracellular functions involve proinflammatory, antimicrobial, oxidant-scavenging, and apoptosis-inducing activities. Its proinflammatory activity includes recruitment of leukocytes, promotion of cytokine and chemokine production, and regulation of leukocyte adhesion and migration. Calprotectin also stimulates innate immune cells via binding to pattern recognition receptors such as Toll-like receptor 4 (TLR4) and receptor for advanced glycation end products (AGER). These receptors modulate vascular inflammation and play an important role in atherosclerosis pathogenesis. Binding to TLR4 and AGER activates the MAP-kinase and NF-kappa-B signaling pathways resulting in the amplification of the proinflammatory cascade and IL-6 secretion. Calprotectin is found in high concentrations at local sites of inflammation or in the serum of patients with inflammatory diseases such as rheumatoid, cystic fibrosis, inflammatory bowel disease, Crohn’s disease, giant cell arteritis, cystic fibrosis, systemic lupus erythematosus, and progressive systemic sclerosis. A transcriptional profiling approach in patients with acute coronary syndromes identified S100A9 as a novel predictor of myocardial infarction [9]. Further studies demonstrated that elevated plasma levels of S100A9 heterodimer predict increased risk of first and recurrent cardiovascular events [10]. Up-regulation of S100A9 heterodimer is involved in atherosclerosis [11]. Thus, S100A9 broadly regulates vascular inflammation and contributes to the biological response to vascular injury [12].

Consequently, an increased S100A9 level at R + 1 could serve as a marker of activation of proinflammatory reactions or damage of endothelial cells due to landing stress. Endothelium is crucial for human vascular integrity and blood vessel function and its damage causes changes in the vascular wall similar to inflammation. In any case, increased levels of this protein can adversely affect cosmonauts’ vessels, which can have long-term consequences.

Analyzing the dynamics of 19 reliably different proteins, we found that most of these proteins are decreased on the first day after landing compared with L-30 (except for the proteins PAI-3 and S100A9). Compared to R + 1, these proteins were found to have increased at R + 7 (excluding apolipoprotein A-II, serotransferrin, insulin-like growth factor binding protein, and mannan-binding lectin serine protease 1), approaching background levels or remaining just below them. Thus, the contents of these proteins in the plasma were restored. However, the concentration of fibulin-1 (Fig. 1 and Additional file 1: Figure S1) on the seventh day after landing was increased and significantly differed from both background and R + 1. Fibulin-1 is a fibronectin fiber. As it is able to bind fibrin, it participates in the organization of extracellular matrix and maintenance of hemostasis. In our data set, concentration of fibrin precursors (fibrinogens) was increased by the first and seventh days after landing (Table 2), followed by fibulin-1.

**Table 2:** Proteins with increased or stable concentration at R + 1

Protein name	Means, fmol/μl			Complement component	Acute phase proteins	Proteases	Protease inhibitors
	L-30	R + 1	R + 7				
**Alpha-1-antichymotrypsin**	2392.5	2411.6	2473.1		+		+
Apolipoprotein B-100	474.9	478.1	415.9				
**Apolipoprotein E**	797.9	914.5	915.8				
**Beta-2-glycoprotein 1**	3367.2	3387.3	3471.1				
**C4b-binding protein alpha chain**	208.1	208.9	211.1				
Carbonic anhydrase 1	55.4	69.9	56.8				
**Ceruloplasmin**	2158.3	2182.9	2292.2		+		
Coagulation factor XI	46.9	49.3	46.2	+		+	
**Complement C2**	108.4	109.5	110.0	+		+	
**Complement C3**	5936.4	5986.7	6141.2	+	+	+	
**Complement C4-B**	1639.5	1651.7	1691.3	+		+	
Complement component C9	215.0	252.3	247.2	+	+		
**Fibrinogen alpha chain**	6781.9	7651.4	7726.8	+	+		
**Fibrinogen beta chain**	419.0	423.5	428.7	+	+		
**Fibrinogen gamma chain**	426.4	480.3	490.1	+	+		
**Fibrinopeptide A**	1469.0	1593.3	1663.9				
**Haptoglobin**	18,033.4	19,230.8	19,612.7		+	+	
Hemoglobin subunit alpha	2628.5	3248.1	2452.5				
Insulin-like growth factor-binding protein complex acid labile subunit	143.3	156.9	148.1				
Leucine-rich alpha-2-glycoprotein	271.2	321.2	308.2				
Lipopolysaccharide-binding protein	121.1	126.8	119.4		+		
MRNA for apolipoprotein E	677.4	790.8	778.8				
**Plasma serine protease inhibitor (PAI-3)**	89.9	91.8	104.7	+			+
**Protein AMBP**	926.0	1007.6	1080.5				+
Protein S100-A9	2.7	6.2	3.8				
Serum paraoxonase/lactonase 3	20.0	20.2	18.8				
Thyroxine-binding globulin	166.9	173.6	171.6				+
**Vitamin D-binding protein**	3222.1	3301.9	3306.5				
**Vitamin K-dependent protein C**	40.4	40.6	41.5	+		+	
**Vitronectin**	325.1	326.4	352.1				

Note: proteins which continue to increase concentration at R + 7 are bold

Analyzing concentrations of blood proteins, we took into consideration that plasma volume is reduced by approximately 10–17% within the first day of spaceflight. Total red blood cell mass decreases by approximately 10% within 1 week in space [5]. Then the amount of proteins is reduced to maintain osmotic homeostasis established under conditions of weightlessness. When the hypovolemic cosmonauts return to upright position in gravity, cardiac stroke volume and arterial pressure falls below pre-flight upright levels [6], and fluid-retaining systems become activated. Compared with pre-flight levels, on the landing day urinary ADH levels increase almost threefold, plasma renin activity increases almost fourfold, plasma aldosterone levels increase by 50%, and plasma atrial natriuretic peptide levels decrease by 33% [13]. Also, to prevent reduction in the extracellular fluid volume the cosmonauts are administered water-salt supplements before landing to activate water retention mechanisms and improve body water balance in the post-landing period [14]. Fluid intake is relatively high on landing day, and urine volume is reduced compared with pre-flight values. Fluid metabolism appears to normalize within 1 week after returning from short-term space flight [13]. Long-term space flights may require longer recovery periods. However, changes in protein concentrations can also be caused by other space flight factors, including emotional stress, circadian rhythm shift and overloads during the landing stage.

Thus, in the early post-flight period blood is ‘diluted’ by extra fluid and this leads to a decrease in the concentration of plasma proteins at R + 1 (Additional file 1: Figure S1). In addition, in this experiment we observed a decrease in concentration of the major protein in the blood plasma, albumin, although this decrease did not rise to the level of significance. Still, it is necessary to resupply proteins in plasma to recover colloid-oncotic pressure. We supposed that synthesis of protease inhibitors such as PAI-3 is activated after space flight to deactivate proteolysis. It was shown that protease inhibitors are activated in response to hypoproteinemia. Hypoproteinemia due to sepsis leads to a 2–5 fold decrease of the activity of endogenous proteinases while the activity of protease inhibitors, alpha 1-antichymotrypsin and an acid-stable inhibitor of trypsin, was found to be higher by 20–30% in blood plasma [15].

Despite the plasma ‘dilution’, it is possible to distinguish proteins that decrease more than others do. Two proteins, cystatin-C and alpha-2-HS-glycoprotein, which are cysteine protease inhibitors, decrease their concentration at R + 1 (by 41.8 and 40% respectively) (Fig. 1). Thus, the dynamics of protease inhibitors is ambiguous, but changes in this system can be clearly traced.

Blood ‘dilution’ due to space flight makes the detection of proteins with increased concentration more difficult. Therefore, proteins whose concentration increased on the first day after landing or did not decrease with the majority of cosmonauts’ blood proteins, although not rising to the level of significance, were of interest. From the total number of identified proteins (125) we analyzed 30 proteins with increased or stable concentration at R + 1 (Table 2).

Using web application AmiGO and DAVID database we performed functional analysis of these proteins. Complement and coagulation cascades are significantly more represented pathways (*p* < 0.05 with consideration of Benjamini-Hochberg correction for multiple comparisons). The complement system is a cascade of proteolytic enzymes. The principal function of the complement system is protection of the host from infection/inflammation by recruiting (chemotaxis) and enhancing phagocytosis by innate immune cells, leading to lysis of the target cells. It is a part of both the innate and adaptive immune responses. The complement system can potentially damage host cells as well, so when the complement system is stimulated by proteases, it could damage endothelium.

Ten proteins with increased or stable concentrations at R + 1 participate in complement and coagulation cascades. Eight of these proteins (Complement C2, C3, C4B, fibrinogen alpha, beta and gamma chain, vitamin K-dependent protein C, and PAI-3) are increased at R + 1 and continue to increase at R + 7 (Table 2). Thus, we can suppose that increased synthesis of these proteins is induced by space flight.

Five proteins of the complement and coagulation cascades (coagulation factor XI, complement C2, C3, C4B, and protein C) are proteases. Thus, five proteases are increased at R + 1 after space flight; all of them are serine-type endopeptidases.

Together with serine proteases, serine protease inhibitors such as α1-antichymotrypsin, protein AMBP, thyroxine-binding globulin, and PAI-3 were increased at R + 1 (Table 2). In addition, proteins participating in negative regulation of endopeptidase activity (vitronectin and complement C3) were increased or unchanged. PAI-3 was increased at R + 1 and continued to increase at R + 7 (Table 1) reaching significance, as detected by ANOVA. Thus, we can suppose that increased synthesis of this protein is induced by space flight.

Thus, a large portion of proteins with decreased or increased concentration at R + 1 were proteases and protease inhibitors. Redistribution of the amounts of proteases and their inhibitors has physiological value. It is necessary to satisfy the requirement of an organism for new proteins. Proteases cleave old proteins and generate amino acids which are used for rapid building of new proteins that are necessary for adaptation processes. It is known that the concentrations of free amino acids (lysine, threonine, valine, leucine, isoleucine, and phenylalanine) are decreased in blood after space flight [16]. Therefore, a blood supply with amino acids produced by proteases represents an acute adaptive reaction. In the first days after spaceflight an organism needs plastic resources (proteins) for muscle tissue reconstruction and possibly blood oncotic pressure recovery.

Concentrations of 9 acute-phase proteins in the plasma were increased in our data set. Seven of them (complement C3, 1-antichymotrypsin, fibrinogen α, β and γ chain, ceruloplasmin, and haptoglobin) continued to increase in concentration by R + 7 (Table 2). Acute-phase proteins are a class of proteins in which plasma concentrations increase (positive acute-phase proteins) or decrease (negative acute-phase proteins) in response to inflammation or injury. Several of them have antiprotease activity. It is worth saying that some negative acute-phase proteins (serotransferrin and alpha-2-HS-glycoprotein) were significantly decreased (Table 1).

A number of studies confirm an increase of acute phase proteins after space flight. Kaur and Simons [17] showed that levels of the lipopolysaccharide binding protein in the plasma of 20 crew members, 3–4 h and 15 days after landing, were increased. Fibrinogen concentration was significantly increased (by 13.2%) on the first day after space flight [18]. Even after short-duration space flights lasting 4–7 days, the phenomenon of fibrinogenemia (an increased level of fibrinogen in the blood) was demonstrated [19]. Stein et al. showed an increased rate of fibrinogen synthesis on the first day after the end of a 16-day flight [20]. However, increased concentration of fibrinogen is observed not only in acute inflammatory processes in the body, but also during stress [21] and at decreased levels of physical activity [22]. It is possible the day of landing is associated with short-term metabolic stress, as suggested by the confirmed increased synthesis of fibrinogen and release of cortisol and interleukin-6 [23].

There are other works confirming our results. After 49-days of spaceflight, increased levels of α-2 globulins, ceruloplasmin, and haptoglobin were demonstrated, whose concentrations were maximal on the 14th day of the re-adaptation period [24]. Significant shifts of β-globulin fractions were observed: transferrin was decreased and C3 and C4 complement factors were increased on the first post-flight day. In our study, similar changes were detected. The authors point out the development of the acute phase response after the end of the flight. This response could be due to both the accelerations from descent and landing as well as the conditions associated with a return to terrestrial gravity.

In all mammals, the synthesis of acute phase proteins is regulated by inflammatory cytokines, such as interleukin-6 (IL-6), interleukin-1 (IL-1), and tumor necrosis factor (TNF). For example, IL-6 plays an important role in the regulation of haptoglobin, fibrinogen, and alpha-1-anti-chymotrypsin. There is evidence that human endothelial cells have increased secretion of IL-6 after exposure to spaceflight conditions [25]. Stein and Schluter found that urinary IL-6 and cortisol excretion were increased on the 1st day of spaceflight, suggesting an acute-phase response to a stress during adaptation to the space environment [26]. On the day of landing, there was also a significant increase in IL-6 levels in urine in some astronauts [27]. However, excretion of cortisol in urine corresponded to the background level, which lowers the probability that emotional stress plays a role in the induction of the acute phase response. The question of whether or not the acute phase response develops after the landing remains to be answered.

The increasing S100A9 concentration could be attributed to induction of IL-6 synthesis. It has been shown that the above-described S100A9 protein is able to induce IL-6 production in human gingival fibroblasts [28] and in human endothelial cells pretreated with AGE albumin [29]. There is evidence that in addition to participating in the inflammatory response, S100A8 and S100A9 proteins are involved in angiogenesis due to their ability to enhance proliferation, migration, and formation of tubular structures by endothelial cells [30]. It is known that angiogenesis is enhanced by inflammation. Thus, the S100A9 protein could be the mechanism of angiogenesis activation occurring in inflammation.

Some of the proteins with increased concentrations at R + 1 also contribute to angiogenesis. Leucine-rich alpha-2-glycoprotein positively regulates proliferation of endothelial cells. Complement C3 stimulates the production of vascular endothelial growth factor (VEGF) and vitronectin positively regulates the VEGF signal pathway. VEGF stimulates angiogenesis, vascular growth, endothelial cells growth, as well as their proliferation and migration, both necessary for angiogenesis. In the tissues of skeletal muscles, the most important factor of angiogenesis is VEGF. The secretion of VEGF from muscle cells is stimulated by various factors including muscle contraction. Therefore, the concentration of VEGF increases markedly in muscle activity, which triggers angiogenesis. Interestingly, our data indicate that during the first day after landing, angiogenesis processes are activated, related to the restoration of muscle mass and the need to ensure their adequate blood supply. In the work [31] it is confirmed that skeletal muscle training has a strong angiogenic effect. It is possible that after space flight the hypotrophic muscles are reloaded and activation of inflammation at a low level occurs. Accordingly, it can be assumed that during the acute period of re-adaptation, an inflammatory response is initiated as activation of pro-inflammatory reactions is physiologically necessary to trigger neovascularization in the muscles that are hypotrophic after landing.

The data obtained by new analysis confirm the involvement of pathways that regulate the activities of proteases, natural immunity, lipid metabolism, and coagulation cascades that were determined in a previous analysis [3]. In addition, the article explains the dynamics of the most extensive group of proteins described in a previous article and whose concentrations recovered rapidly to their pre-flight levels [3]. It was assumed that changes in the subjects’ blood concentrations are transitory and may be attributable to the influence of the final stage of the flight, including such factors as emotional stress and overloads during the landing stage. In the light of new data, it is believed that the dynamics of these proteins are also associated with changes in the volume of circulating plasma.

## Conclusion

Reduction of circulating plasma volume during space flight, and subsequent activation of fluid retention at the final stage of flight - affect the changes in plasma protein concentrations in the first days after landing. Using the ANOVA approach, it was revealed that only 1 protein reliably responded to the space flight conditions described herein - S100A9. This protein plays an important role in the functioning of endothelium and can serve as a marker for the activation of inflammatory reactions. There are increases in the concentrations of the proteins of complement and the coagulation cascade, as well as an increase in the acute phase response, in the blood of cosmonauts on the first day after landing. Most of these proteins’ concentrations continue to increase by the 7th day after landing. Similar dynamics are observed for proteases and their inhibitors. Thus, there is a shift in proteolytic blood systems, which is necessary for the restoration of muscle tissue and maintenance of oncotic homeostasis. We assume that during the acute period of cosmonauts’ re-adaptation, the load of hypotrophic muscles under conditions of terrestrial gravity initiates an inflammatory response and activation of angiogenesis processes. Consequently, using the proteomic method of studying the blood of cosmonauts to expand the understanding of the mechanisms of the adaptive process will perhaps serve as the basis for the development of preventive measures.

## Additional file

Additional file 1 : **Figure S1.** Box & Whisker plots of 19 proteins with statistically significant differences between points of blood collection. (DOCX 521 kb)

## Abbreviations

ANOVA: Analysis of variance
CVS: Cardiovascular system
PAI-3: Plasma serine protease inhibitor
VEGF: Vascular endothelial growth factor
