# Supplementary material for: The molecular mechanisms driving physiological changes after long duration space flights revealed by quantitative analysis of human blood proteins
Source: BMC Med Genomics. 2019 Mar 13;12(Suppl 2):45. doi: 10.1186/s12920-019-0490-y (PMC6416832; doi:10.1186/s12920-019-0490-y)
Supplement: Supplementary file 1 — : Figure S1. Box & Whisker plots of 19 proteins with statistically significant differences between points of blood collection. (DOCX 521 kb) [file 12920_2019_490_MOESM1_ESM.docx]

**Additional file 1**

**Figure S1. Box & Whisker plots of 19 proteins with statistically significant differences between points of blood collection.**
